# *Escherichia* coli surface display for the selection of nanobodies

**DOI:** 10.1111/1751-7915.12819

**Published:** 2017-08-03

**Authors:** Valencio Salema, Luis Ángel Fernández

**Affiliations:** ^1^ Department of Microbial Biotechnology Centro Nacional de Biotecnología (CNB) Consejo Superior de Investigaciones Científicas (CSIC) Madrid Spain

## Abstract

Nanobodies (Nbs) are the smallest functional antibody fragments known in nature and have multiple applications in biomedicine or environmental monitoring. Nbs are derived from the variable segment of camelid heavy chain‐only antibodies, known as VHH. For selection, libraries of VHH gene segments from naïve, immunized animals or of synthetic origin have been traditionally cloned in *E. coli* phage display or yeast display systems, and clones binding the target antigen recovered, usually from plastic surfaces with the immobilized antigen (phage display) or using fluorescence‐activated cell sorting (FACS; yeast display). This review briefly describes these conventional approaches and focuses on the distinct properties of an *E. coli* display system developed in our laboratory, which combines the benefits of both phage display and yeast display systems. We demonstrate that *E. coli* display using an N‐terminal domain of intimin is an effective platform for the surface display of VHH libraries enabling selection of high‐affinity Nbs by magnetic cell sorting and direct selection on live mammalian cells displaying the target antigen on their surface. Flow cytometry analysis of *E. coli* bacteria displaying the Nbs on their surface allows monitoring of the selection process, facilitates screening, characterization of antigen‐binding clones, specificity, ligand competition and estimation of the equilibrium dissociation constant (K_D_).

## Introduction

**Figure 1:**
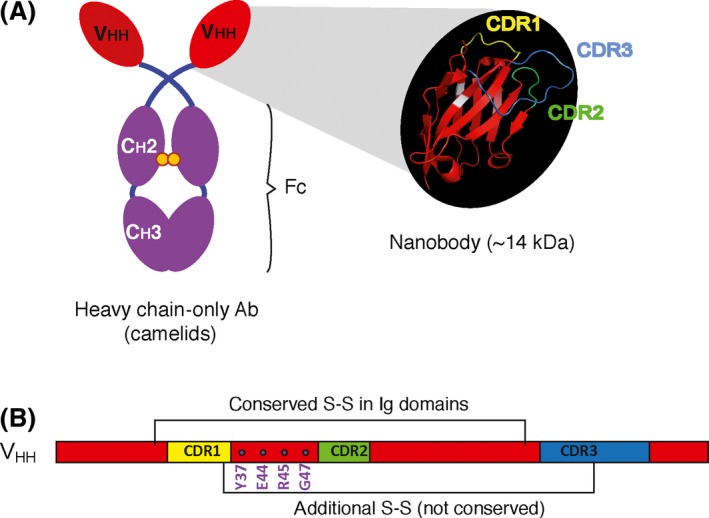
Structure of camelid heavy chain‐only antibodies and nanobodies. (A) Schematic representation of a heavy chain‐only Ab with the structure of the VHH domain (Nb) highlighted. The complementarity‐determining regions (CDRs) are labelled in different colours: CDR1 in yellow, CDR2 in green, CDR3 in blue and the framework regions (FR) are indicated in red. (B) The linear structural diagram of a VHH domain, indicating CDRs, disulfide bonds (S‐S) in the molecule and positions of framework region 2 of VHHs that contain more hydrophilic amino acids (e.g. Y37, E44, R45, G47) compared with conventional VHs (e.g. V37, G44, L45, W47). The conserved canonical S‐S in Ig domains and the additional non‐conserved S‐S found in many Nbs are also illustrated.

Nanobodies (Nbs) are the smallest, intact antigen‐binding fragments derived from a functional immunoglobulin (Ig). Nbs are recombinant single domain antibody (Ab) fragments with a molecular weight of ~14 kDa and ~2–4 nm in size. They comprise the variable domain of a heavy chain‐only antibody (HCAb; Fig. 1A), which was discovered in the serum of camelids in the early 1990s (Hamers‐Casterman *et al*., 1993), and are likely an outcome of adaptive changes occurring in conventional Abs within the Camelidae lineage, playing a role in the immune response of these animals (Nguyen *et al*., 2002; Flajnik *et al*., 2011; Muyldermans and Smider, 2016). HCAbs lack light chains, and thus, antigen recognition is possible solely through the variable domain of the heavy chain, referred to as VHH for VH of HCAbs. Although Nbs and VHHS are often used interchangeably, it is generally accepted that VHH refers to the gene, or to the polypeptide with unknown specificity, and Nb to the recombinant, purified antigen‐specific polypeptide.

Nbs have naturally acquired important adaptations to remain soluble and functional in the absence of the associated light chain variable domain. They have evolved long complementarity‐determining regions (CDRs, Fig. 1B) capable of adopting novel conformations for antigen recognition, subtle amino acid adaptations including substitutions of conserved hydrophobic residues in classical VHs (V37, G44, L45 and F47/W47) for more hydrophilic amino acids (Y37/F37; E44/Q44; R45/C45; G47/R47/L47/S47; Muyldermans, 2013; Muyldermans and Smider, 2016), which confer them strict monomeric behaviour, reversible folding properties, resistance to proteolysis and thermal degradation when compared with the VH from conventional antibodies (van der Linden *et al*., 1999; Muyldermans *et al*., 2001; Dumoulin *et al*., 2002). Furthermore, Nbs often contain, besides the canonical disulfide bond of Ig domains, an extra disulfide bond connecting CDR3 and CDR1 (in camels) or CDR3 with CDR2 (in llamas; Fig. 1B) that assist in stabilizing the conformation of these CDRs and the overall stability of the domain (Govaert *et al*., 2012). The small size and long CDRs of Nbs allow them to successfully target enzymatic clefts located within concave epitopes of enzymes and proteases (Lauwereys *et al*., 1998; Conrath *et al*., 2001; Kromann‐Hansen *et al*., 2016), and conserved inner regions of surface proteins from pathogens, frequently less accessible to the larger conventional Abs (Stijlemans *et al*., 2004, 2011; Rossey *et al*., 2017). Nbs have been used as stabilization agents in crystallographic studies (Ring *et al*., 2013; Hassaine *et al*., 2014; Pardon *et al*., 2014) and as molecular probes in cell biology for visualization of proteins in living cells (Helma *et al*., 2015). In addition, they share high sequence identity with human VH sequences of family 3, which opens the possibility of their use in human therapy and in *in vivo* diagnosis (Vaneycken *et al*., 2011; Chakravarty *et al*., 2014; Muyldermans and Smider, 2016; Steeland *et al*., 2016). The short serum half‐life of Nbs due to renal clearance may limit the efficacy of monomeric Nbs in therapeutic applications, but this has been largely overcome by generation of bispecific Nbs recognizing long‐lived serum proteins (e.g. albumin) and the therapeutic target, thus resulting in increased serum half‐lives (Tijink *et al*., 2008; Vosjan *et al*., 2012). In addition, the stability and small size of Nbs make them ideal candidates for targeting intracellular proteins and show great potential as intrabodies (Lobato and Rabbitts, 2003; Blanco‐Toribio *et al*., 2010; Bouchet *et al*., 2011; Van Impe *et al*., 2013; Bethuyne *et al*., 2014; Fulcher *et al*., 2016; Moutel *et al*., 2016). Other applications of Nbs have been recently reviewed (Bruce *et al*., 2016).

Besides these advantages, Nbs can be easily expressed in *E. coli*, where they are produced as soluble proteins with incorporated affinity tags for purification (Arbabi‐Ghahroudi *et al*., 2005; Salema and Fernández, 2013). Higher production levels can be obtained from high‐density yeast cultures, like *Pichia pastoris* and *Saccharomyces cerevisiae* (Frenken *et al*., 2000; Holliger, 2002), or in plants (Rajabi‐Memari *et al*., 2006; De Meyer *et al*., 2015).

Libraries of VHHs are commonly generated through camel immunization, often dromedaries (*Camelus dromedarius*) or llamas (*Lama glama*; Arbabi Ghahroudi *et al*., 1997; Conrath *et al*., 2001; Koch‐Nolte *et al*., 2007; Hassanzadeh‐Ghassabeh *et al*., 2011), but these may also be obtained from naïve (non‐immune) animals or be from synthetic origin by *in vitro* CDR randomization (Monegal *et al*., 2009; Olichon and de Marco, 2012; Moutel *et al*., 2016). In most cases, the VHHs are cloned into a vector that allows their expression on the surface of a biological entity, usually a bacteriophage or a microbial cell (i.e. yeast, bacteria). Similar to other Ab fragments, the library of bacteriophage or microbial cells displaying the Nbs is then incubated with the target antigen either immobilized, in the soluble biotinylated form, or expressed on cells, for selection (Hoogenboom, 2005). Phages or microbial cells binding the target antigen are isolated and amplified, resulting in the enrichment of high‐affinity binders expressing the selected Nbs.

## Phage display

**Figure 2:**
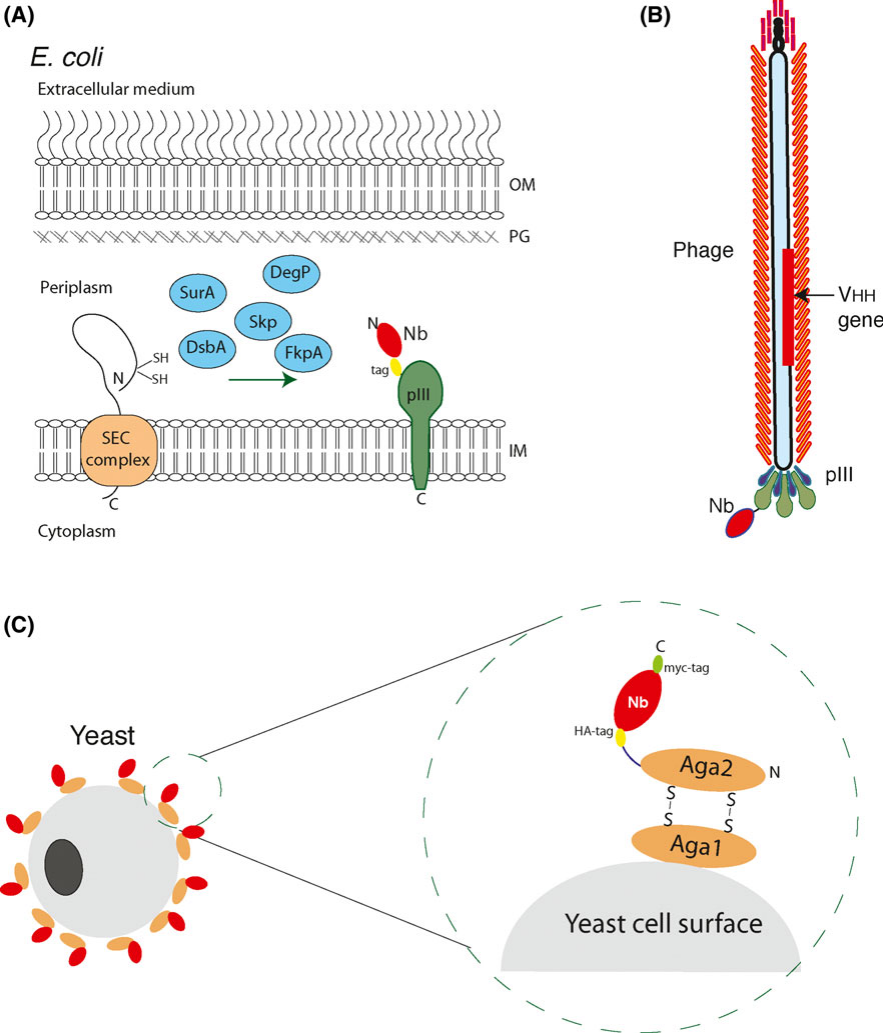
Phage display and yeast display of nanobodies. (A) Expression of Nbs fused to the minor coat protein III (pIII) of filamentous phages in the periplasm of *E. coli*. The fusion protein is exported to the periplasm with a N‐terminal signal peptide (e.g. PelB) through the Sec complex. The Nb domain is exposed to the periplasm and is tethered to the inner membrane (IM) of *E. coli* by pIII. An epitope tag (e.g. HA, myc) is usually included between Nb and pIII moieties. Periplasmic chaperones (e.g. SurA, Skp, FkpA, DegP) and disulfide bond catalysts (e.g. DsbA) assist folding of the Nb domain in the periplasm. The *E. coli* inner membrane (IM), peptidoglycan (PG) and outer membrane (OM) are also indicated. (B) Scheme of a filamentous phage–Nb particle assembled upon infection of *E. coli* cells expressing Nb‐pIII fusions with a helper phage. The Nb‐pIII fusion is displayed at the tip of the filamentous phage capsid (usually one copy per phage), which contains the phagemid DNA with the encoding VHH gene. (C) Scheme of the display of Nbs on a yeast cell fused to the C‐terminus of the yeast agglutinin receptor (Aga2) present on the yeast cell surface (enlarged). The Aga2‐Nb fusion follows the secretory pathway of yeast cells from the endoplasmic reticulum (ER) to the plasma membrane. Epitope tags flanking the Nb domain are also indicated (e.g. HA, myc).

Phage display is a powerful and robust technology that allows display of Nbs (and other Ab fragments) on the surface of filamentous bacteriophages infecting *E. coli* through the F pilus, such as M13 or f1 (Rakonjac *et al*., 2011; Verheesen and Laeremans, 2012; Frenzel *et al*., 2016). In the most common configuration, the VHHs are cloned in a phagemid vector as fusions to the minor phage coat protein 3 (pIII; Fig. 2A; Qi *et al*., 2012). The cloned VHH is also in frame with an N‐terminal signal peptide driving insertion of the protein fusion into the bacterial inner membrane (IM) of *E. coli* (Thie *et al*., 2008). Upon IM insertion, the Nb domain of the Nb‐pIII fusion faces the bacterial periplasm, where protein chaperones and specialized disulfide bond (Dsb) catalysts assist on the folding of the Ig V domains (Fig. 2A; Kadokura *et al*., 2003; Heras *et al*., 2009; Schlapschy and Skerra, 2011; Bodelón *et al*., 2013). The Nb‐pIII fusions are then assembled into phage particles (carrying the phagemid DNA) by infection of bacteria with a ‘helper’ phage that provides all proteins needed for phage replication but has a defective origin of packaging (Rakonjac *et al*., 2011). A scheme illustrating the display of the Nb into the final phage particle is shown in Fig. 2B. The high phage titres that are obtained from infected *E. coli* cultures (≥10^11^ phages ml^−1^) ensure a good representation of VHHs found in the library, even when large libraries with > 10^9^ clones are utilized (e.g. from naïve and synthetic origins). Nonetheless, representation of VHHs in large phage libraries is also limited by intrinsic *E. coli* host factors like the capacity of the helper phage to initially infect a sufficient number of bacteria (> 10^9^) in the culture that will produce the phage particles. In addition, phage particles display a maximum of one copy of the Nb‐pIII fusion, with the large majority of phage particles not displaying any fusion unless a helper phage mutant △pIII is used (Rondot *et al*., 2001; Soltes *et al*., 2003). In all cases, phagemid particles produced from these cultures are concentrated by precipitation, titred with fresh *E. coli* cultures and maintained in high‐titre stocks > 10^13^ colony‐forming units (CFU) ml^−1^. These phage stocks are subjected to ‘panning’ to enrich for antigen‐binding clones. Most often, this is achieved by incubating phages with the purified antigen, either immobilized onto a plastic surface or in a soluble form covalently labelled with biotin. Due to the ‘sticky’ nature of filamentous phages, extensive and stringent washing conditions need to be used to remove unbound phage particles. Lastly, the bound phages are recovered by elution using acid or alkaline buffers, limited proteolysis, or by competition with soluble antigen, ligand or specific mAb binding an epitope of interest on the antigen (Hoogenboom, 2002). Isolated phage particles are then used to infect *E. coli* for amplification and eventual analysis of antigen binding by ‘phage ELISA’ using secondary antibodies against the major phage coat protein. Multiple examples of high‐affinity Nbs selected by phage display using purified antigens can be found in the literature; just a few recent examples are cited here (Bethuyne *et al*., 2014; Lo *et al*., 2014; Kazemi‐Lomedasht *et al*., 2015; Nguyen *et al*., 2015; Zhou *et al*., 2016; Rossey *et al*., 2017). However, despite its widespread use, phage display suffers some limitations. These include the need of extensive manipulation for the production of phage stocks (i.e. infection of bacteria with helper phage, amplification by re‐infection), the low levels of Nb display and the high background binding of phage particles, which makes selections against complex non‐purified antigens (e.g. intact cells and tissues) more challenging (Siva *et al*., 2008; Even‐Desrumeaux *et al*., 2014; Pavoni *et al*., 2014).

## Yeast display

Cell surface display systems are alternative methods of protein display and are effective tools for Ab or Nb selection and engineering by directed evolution (e.g. affinity maturation; Pardon *et al*., 2014; Galan *et al*., 2016). The basic principle is analogous to phage display, providing a physical linkage between the displayed polypeptide anchored in the cell surface and its encoding gene inside the cell. Cell surface display systems have been developed for different host microorganisms, including bacteria and yeast cells. General benefits of these systems versus phage display are their reduced background and culture manipulation, higher surface display level and the possibility of utilizing high‐throughput methods such as fluorescence flow cytometry for the selection of binders and characterization of their antigen affinity and specificity (Gai and Wittrup, 2007). Yeast display system, originally described in *Saccharomyces cerevisiae* (Boder and Wittrup, 1997, 2000; Feldhaus and Siegel, 2004; Chao *et al*., 2006) and also developed in the methylotrophic yeast *Pichia pastoris* (De Schutter and Callewaert, 2012), has been applied in Ab engineering. This system utilizes the α‐agglutinin adhesion receptor found on the surface of yeast cells for protein display (Fig. 2C). The receptor consists of two secreted proteins, Aga1 and Aga2, which are covalently bound to the yeast cell wall. Aga1 attaches to β‐glucans, whereas Aga2 is bound to Aga1 by two disulfide bonds. The Ab fragment to be displayed is fused in frame to Aga2 C‐terminus in a plasmid vector with a regulated promoter (*GAL1*). The protein fusion also includes two epitope tags (e.g. HA‐ and myc‐tag) flanking the Ab fragment, which enable its detection and surface display using specific monoclonal Abs (mAbs) by fluorescence flow cytometry. The use of a eukaryotic secretory pathway, coupled with protein folding and quality control systems of yeasts, is thought to contribute to the correct folding of more complex mammalian Ab or Ab fragments on yeast cells. The display level on the yeast cell is variable but has been estimated about 3–5 × 10^4^ fusions per cell for single‐chain variable fragments (scFv; Chao *et al*., 2006; Gai and Wittrup, 2007; Pepper *et al*., 2008). The potential intrinsic avidity of these elevated display levels is counteracted by the power of fluorescence‐activated cell sorting (FACS). By staining with both soluble biotinylated antigen and the epitope‐tag mAb, and subsequent incubation with secondary fluorescence‐labelled reagents (e.g. streptavidin, anti‐mouse IgG), the yeast cells can be sorted by FACS according to the level of antigen binding and Ab fusion displayed on the surface. Avidity effects are limited using monomeric antigen in solution, but can be increased with multimeric antigens or by preloading tetrameric streptavidin with the biotinylated antigen prior to its incubation with yeast cells (Chao *et al*., 2006). A clear advantage of yeast display is the possibility to characterize the equilibrium dissociation constant (K_D_) of selected Ab clones with a titration curve of the fluorescence signals obtained by flow cytometry of yeast cells with different concentrations of the labelled antigen (Gai and Wittrup, 2007), thus avoiding the need of expression and purification of the soluble Ab fragments to have an estimation of their affinity constants.

Yeast display selections often utilize relatively small libraries of ~10^6^–10^7^ clones, either obtained after antigen immunization or by mutagenesis of a pre‐existing clone for affinity maturation procedures (van den Beucken *et al*., 2003; Colby *et al*., 2004; Siegel, 2009; Koide and Koide, 2012). Larger Ab libraries (e.g. ~10^9^ clones from naïve origin) have also been constructed and screened for antigen‐binding clones (Yeung and Wittrup, 2002; Feldhaus *et al*., 2003; Gai and Wittrup, 2007). However, large libraries are more difficult to generate in yeast than in *E. coli* due to the lower transformation efficiency of yeast cells. Also, yeast cultures require longer times for growth, and reach cellular densities of *c*. 2 × 10^7^ CFU ml^−1^ at late exponential phase, which are much lower than those obtained with *E. coli* cultures (*c*. 1 × 10^9^ CFU ml^−1^). Hence, representation of naïve libraries requires growth of large yeast culture volumes and the concentration of yeast cells (Chao *et al*., 2006). The bigger size of yeast cells than bacteria makes difficult the handling of liquid suspensions concentrated to above 10^9^ CFU ml^−1^, whereas *E. coli* is easily concentrated to ~10^11^ CFU ml^−1^ (e.g. for electroporation). Therefore, the use of an *E. coli* display system that could combine the major advantages of the phage and yeast display systems would be highly beneficial.

## *E. coli* display

### The outer membrane barrier

*Escherichia coli* is the most commonly used bacterial host for expression and engineering of Ab fragments, although other bacteria, especially some Gram‐positive species, have been used for display of Ab fragments and Nbs (Krüger *et al*., 2002; Pant *et al*., 2006; Kronqvist *et al*., 2008; Fleetwood *et al*., 2013). For instance, an immune library of VHHs of *c*. 10^7^ clones was displayed on *Staphylococcus carnosus* cell surface fused to the anchoring domain of protein A, and screened by FACS for Nbs binding GFP (Fleetwood *et al*., 2013). One likely advantage with the use of Gram‐positive bacteria is their rather simple cell envelope with a single cell membrane, which simplifies translocation of the Nbs to the surface. Also, Gram‐positive bacteria have comparative higher tolerance to mechanical stress due to a thicker cell wall, which may result in higher viability during selection and cell sorting. Nonetheless, *E. coli* is a more suitable microorganism for the cloning, amplification and maintenance of large Nb repertoires owing to its high transformation efficiency, the stability of the cloned DNA in laboratory *E. coli* strains, its fast and simple growth and the availability of multiple well‐validated protein expression and secretion systems. The larger size of *E. coli* bacteria than phages allows the direct use of flow cytometry‐based methods for selection, screening and characterization. Also, *E. coli* bacteria enable higher display levels and are less sticky than phages, which results in lower background levels. Lastly, *E. coli* bacteria can be grown and concentrated to sufficiently high densities (see above) to represent large naïve and synthetic libraries.

Despite the apparent beneficial properties of *E. coli*, the development of effective *E. coli* display systems for Ab/Nb fragments has not been straightforward. This is because *E. coli*, being a Gram‐negative bacterium, has a cell envelope with two biological membranes: the cytoplasmic inner membrane (IM) and the outer membrane (OM; Bos *et al*., 2007; Delcour, 2009). These membranes are separated by the periplasmic space, containing a peptidoglycan layer and a variety of transport proteins, enzymes and protein‐folding chaperones that participate, among other processes, in peptidyl‐prolyl cis/trans‐isomerization (e.g. FkpA, SurA) and catalysis of disulfide bridges and their isomerization (e.g. DsbA, DsbC; Kadokura *et al*., 2003; Merdanovic *et al*., 2011). The presence of the OM has been the major obstacle hindering the development of effective display methods for Ab selection in *E. coli*. In order to be displayed on the surface, the polypeptides produced in the cytoplasm have to translocate through the IM, traverse the periplasm, be inserted into the OM and expose the displayed polypeptide region of interest to the extracellular milieu (Dalbey and Kuhn, 2012). Translocation of Nb/Ab fragments to the periplasm using N‐terminal signal peptides of the Sec pathway was solved in phage display vectors (Thie *et al*., 2008; Tsirigotaki *et al*., 2017). Once in the periplasm, chaperones such as DsbA, DsbC, FkpA, SurA and Skp assist correct folding, disulfide bond formation and prolyl *cis*/*trans*‐isomerization of the Ig domains (Bodelón *et al*., 2013). It is known that overexpression of these chaperones increases the production of functional Ab fragments and Nbs in the periplasm of *E. coli* (Schlapschy *et al*., 2006; Friedrich *et al*., 2010; Schlapschy and Skerra, 2011; Shriver‐Lake *et al*., 2017). Hence, the surface display of functional Nb/Ab fragments on *E. coli* requires an OM protein anchor allowing translocation of folded Ig V domains across the OM from the periplasm.

As an alternative to OM display, a periplasmic display method was developed for the selection of Ab libraries on *E. coli*, named ‘anchored periplasmic expression’ (APEx; Harvey *et al*., 2004; Mazor *et al*., 2007, 2008). In APEx, Ab fragments and full‐length IgGs are expressed in the periplasm of *E. coli* tethered to the IM with a chimeric lipoprotein anchor. As the Abs in APEx are not actually displayed on the bacterial surface, access of the labelled antigen for FACS requires permeabilization of the OM to generate spheroplasts of bacteria. But spheroplasts are highly sensitive to lysis, thus restricting the selection conditions that can be used, impeding their direct growth on plates after cell sorting and forcing the amplification, subcloning of the selected Ab genes after every selection cycle, all of which lead to increased manipulation.

### *E. coli* display with outer membrane proteins and surface appendages

Some earlier work reported the surface display of scFv fragments on *E. coli* cells fused to the C‐terminus of the chimeric lipoprotein Lpp‐OmpA’ (Francisco *et al*., 1993), which consists of the N‐terminal signal peptide and the first nine amino acids of the mature lipoprotein Lpp fused to residues 46–159 of OmpA. This truncated fragment of OmpA comprises the first six β‐strands and a portion of the 7th β‐strand of its eight‐stranded β‐barrel (Pautsch and Schulz, 2000) and therefore lacks the characteristic stability of native β‐barrel outer membrane proteins (OMPs; Koebnik *et al*., 2000). This fact may have contributed to the reported OM leakage and cellular toxicity of Lpp‐OmpA’ fusions (Georgiou *et al*., 1996; Stathopoulos *et al*., 1996), which could induce a significant growth bias of clones in libraries. In fact, the Lpp‐OmpA’ fusion was only used in combination with FACS for affinity maturation of preselected scFv clones displayed on *E. coli* (Daugherty *et al*., 1998, 1999), but not for the selection of clones with novel specificities from diverse libraries (either immune or naïve). Native β‐barrel OMPs from *E. coli* and other Gram‐negative bacteria were also analysed for OM anchoring. Interestingly, periplasmic transport of β‐barrel OMPs uses the same set of periplasmic protein chaperones that participate in the folding of Ig domains (Bos *et al*., 2007; Bodelón *et al*., 2013). Porins (e.g. OmpA, OmpC, OmpX, LamB) and protein subunits of fimbrial or flagellar appendages (e.g. FimA, FimH, FliC, FliD) were used as fusion partners for the surface display of peptides and small polypeptides (Lu *et al*., 1995; Sousa *et al*., 1996; Georgiou *et al*., 1997; Klemm and Schembri, 2000; Lee *et al*., 2003; Bessette *et al*., 2004; Majander *et al*., 2005; Rice *et al*., 2006; Munera *et al*., 2007), but none of these systems tolerate the transport of folded Ig domains.

### *E. coli* display with autotransporters and the intimin–invasin proteins

The limitations of the above systems led us to explore alternative *E. coli* display systems for the selection of Nbs (and perhaps other Ab fragments). An important clue came from the study of a large family of proteins secreted by Gram‐negative bacteria called autotransporters (ATs; Henderson *et al*., 2004; Nicolay *et al*., 2015). Classical ATs contain within their sequence a C‐terminal β‐barrel, comprising of 12 antiparallel β‐strands and an internal α‐helix (Oomen *et al*., 2004; van den Berg, 2010; Gawarzewski *et al*., 2014), which acts as a ‘helper’ domain assisting the translocation of the N‐terminal portion of the polypeptide across the OM. The ‘passenger’ N‐domain is the actual part secreted to the medium and usually fold as long β‐helix rod (Leo *et al*., 2012; van Ulsen *et al*., 2014). ATs were originally thought to have self‐translocation capacity across the OM (Pohlner *et al*., 1987; Henderson *et al*., 1998), having an internal pore in the β‐barrel for passenger translocation (Klauser *et al*., 1992; Oomen *et al*., 2004), or forming a protein translocation channel by oligomerization of β‐barrels (Veiga *et al*., 2002). However, compelling evidence supports that the β‐barrel of ATs acts as a signal tethering the polypeptide to the conserved β‐barrel assembly machinery (BAM), or to a related translocation and assembly module (TAM) complex (Ieva and Bernstein, 2009; Sauri *et al*., 2009; Rossiter *et al*., 2011; Gruss *et al*., 2013).

The BAM is a large protein complex in the OM that assists the insertion of β‐barrel proteins in the OM (Knowles *et al*., 2009; Hagan *et al*., 2011; Han *et al*., 2016). The BAM complex associates with the amphipathic β‐strands of unfolded OMPs in the periplasm, assisting the folding of the β‐barrel and its simultaneous insertion in the lipid bilayer of the OM (Noinaj *et al*., 2017). In the case of ATs, the passenger domain appears to be secreted concomitantly with the folding of the β‐barrel (Ieva and Bernstein, 2009), using a translocation pore transiently formed between the β‐barrels of the AT and that of BamA/Omp85 subunit of BAM/TAM complexes (Gruss *et al*., 2013; Noinaj *et al*., 2017). Although the large native β‐helix rod passengers of ATs need to have a partially unfolded conformation for their translocation (Jong *et al*., 2007), we observed that folded Ig domains fused to the β‐barrel of various ATs were efficiently translocated to the surface of *E. coli* cells (Veiga *et al*., 2004; Marín *et al*., 2010). Importantly, an AT‐related family of adhesins, the intimin and invasin family, originally found in enteropathogenic *E. coli* (EPEC), enterohemorrhagic *E. coli* (EHEC) and *Yersinia pseudotuberculosis* and *Y. enterocolitica* strains, naturally have passenger domains based on repeats of Ig‐like domains that are translocated to the bacterial surface (Tsai *et al*., 2010; Leo *et al*., 2015; Sadana *et al*., 2017). Intimin–invasin proteins are similar to ATs but have an opposite topology, with the N‐terminal signal peptide followed by a 12‐stranded β‐barrel with an internal peptide segment (Fairman *et al*., 2012) that connects to a surface‐exposed passenger C‐terminal region comprising repeats of Ig‐like and lectin domains (Hamburger, 1999; Kelly *et al*., 1999; Luo *et al*., 2000; Sadana *et al*., 2017). Work from our laboratory demonstrated that the native Ig‐like domains of intimin fold and form a disulfide bridge catalysed by DsbA in the periplasm prior to their translocation (Bodelón *et al*., 2009). This study also showed that the intimin β‐barrel domain requires the BAM complex for OM insertion, clearly suggesting that they share a similar mechanism of secretion with ATs (Bodelón *et al*., 2009). The capacity to translocate folded Ig domains by intimin and ATs prompted us to investigate and compare these β‐barrel domains for the surface display and selection of Nb libraries in *E. coli* (Salema *et al*., 2013).

### Intimin fusions are optimal for *E. coli* display of Nbs

**Figure 3:**
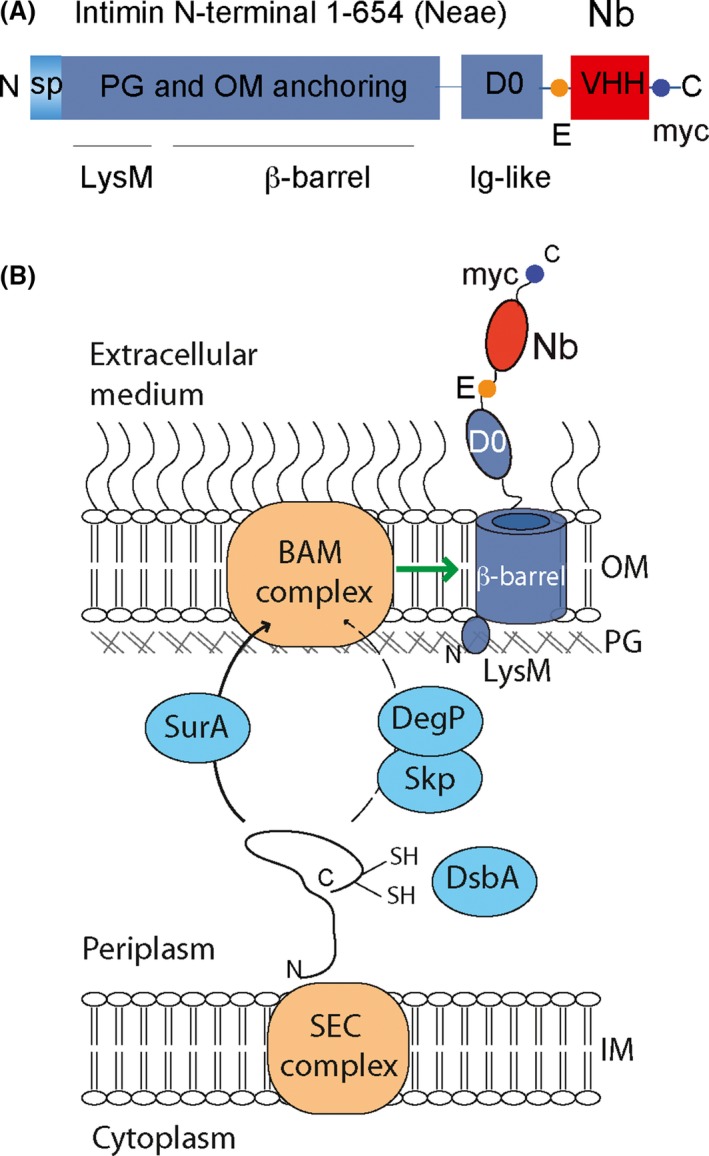
Structure of Neae‐VHH fusions and their secretion mechanism in *E. coli* display of nanobodies. (A) Linear representation of the polypeptide encoded by Neae‐VHH fusions for *E. coli* display. The intimin fragment Neae (residues 1–654) comprises the N‐terminal signal peptide (sp), LysM domain for PG binding, β‐barrel for OM‐anchoring and exposed D0 Ig‐like domain. The VHH encodes the Nb. The Neae‐VHH fusion also contains the E‐tag and myc‐tag epitopes flanking the VHH. (B) The fusion polypeptide expressed in the bacterial cytoplasm is translocated across the IM via the Sec complex. In the periplasm, chaperones assist folding of the Nb and transport of the intimin β‐barrel domain to the BAM complex in the OM. The BAM complex is responsible for the folding and insertion of the intimin β‐barrel in the OM and participates in the translocation of the D0 and Nb domains to the bacterial surface. The epitopes E‐ and myc‐tags are used for the immunodetection of Nb displayed on *E. coli* surface by flow cytometry.

Heterologous peptides and small polypeptides had been previously displayed on *E. coli* and other Gram‐negative bacteria by replacing the native passenger domains of ATs and intimin (Klauser *et al*., 1990; Valls *et al*., 2000; Wentzel *et al*., 2001; Jose and Meyer, 2007). For Nb display, we constructed a plasmid vector for cloning the VHHs in frame with the N‐terminal fragment of EHEC intimin (Neae; residues 1–654) comprising the β‐barrel domain and the first Ig‐like domain called D0 (Salema *et al*., 2013). This vector, called pNeae2, carries the *lac* promoter (Plac) for induction of the fusion protein with isopropylthio‐β‐D‐galactoside (IPTG), includes restriction sites commonly used in phagemids for the cloning of VHHs (*Sfi*I and *Not*I), and epitope tags (E‐tag and myc‐tag) flanking the VHH to detect expression of the fusion protein on *E. coli* surface using commercial mAbs (Fig. 3A and B). We used a similar vector, called pHEA, for fusion of VHHs to the C‐terminal β‐barrel domain of EHEC AT EhaA (C‐EhaA; residues 989–1327), which is well expressed in *E. coli* (Wells *et al*., 2008; Marín *et al*., 2010). Although both types of protein fusions were displayed on *E. coli* surface, higher display levels of Nbs were obtained with intimin fusions, both when individual clones and an immune library of VHHs were compared (Salema *et al*., 2013). Similarly, antigen‐binding signals determined by fluorescence flow cytometry of bacteria displaying Nbs of defined specificity were higher with intimin Neae fusions than with AT C‐EhaA fusions. Upon plasmid induction, *E. coli* bacteria carrying pNeae2‐VHH derivatives displayed ~8000 Nb molecules on their surface. In addition, selection of antigen‐binding clones from immune libraries was more efficient in bacteria displaying intimin Neae fusions than in bacteria displaying C‐EhaA fusions, requiring less number of selection cycles (i.e. two versus four cycles; Salema *et al*., 2013). Therefore, the Neae fragment of intimin proved to be an effective system for display and selection of Nbs in *E. coli*, showing higher expression levels and antigen‐binding signals.

Albeit most *E. coli* laboratory strains could potentially be used with Neae display, we chose two *E. coli* K‐12 strains with deletions in the *fim* operon encoding type 1 fimbriae to avoid the possible interference of these expressed proteinaceous filaments on *E. coli* surface with the binding of antigens to the OM (Hasman *et al*., 1999). We have successfully employed the reference K‐12 strain MG1655 (Blattner *et al*., 1997) with deletion of *fimA*‐*fimH* (called AAEC072 or EcM1; Blomfield *et al*., 1991; Salema *et al*., 2013), and the high‐efficiency cloning strain DH10B‐T1R that naturally lacks the *fim* operon (Durfee *et al*., 2008). Both strains showed similar levels of Nb display on their surface, without any deleterious effects on bacterial growth and viability upon induction.

### Selection of Nbs by *E. coli* display using magnetic cell sorting (MACS)

**Figure 4:**
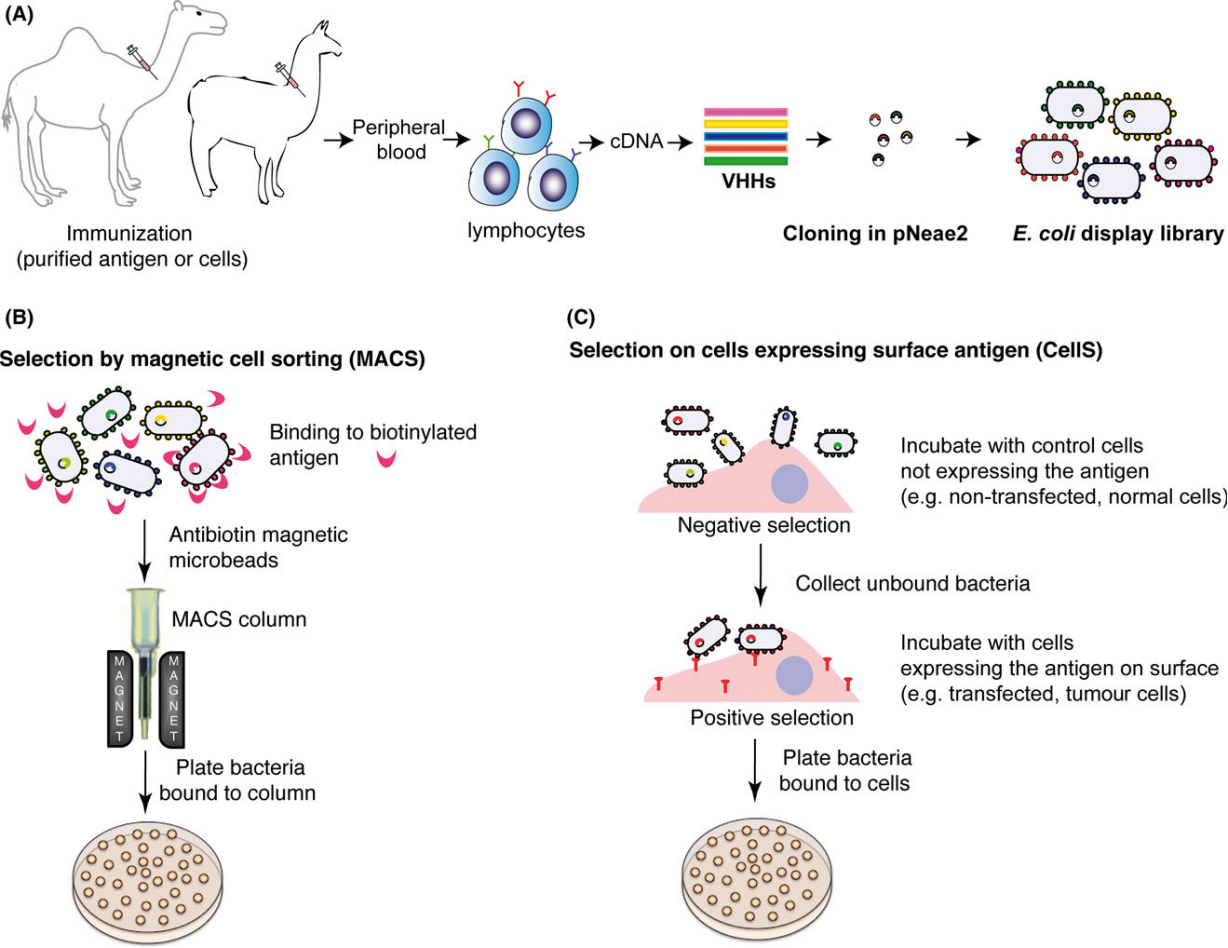
Generation of *E. coli* display immune libraries and their selection by magnetic cell sorting (MACS) and on mammalian cells (CellS). (A) Scheme of the generation of an *E. coli* display immune library of Nbs. Animals (e.g. dromedaries or llamas) are immunized with either purified antigen or cells expressing the target antigen. After immunization, the VHH gene segments are amplified from peripheral blood lymphocytes by RT‐PCR, cloned into plasmid vector pNeae2 and induced on *E. coli* for surface display. (B) Selection of an *E. coli* display library using MACS. *Escherichia coli* bacteria are incubated with the biotinylated antigen and antibiotin magnetic beads. Bacteria with bound antigen are captured in an iron column held in a magnet. Elution of bound bacteria is carried out with fresh LB media upon column removal from the magnet and grown by plating. (C) Selection of an *E. coli* display library on mammalian cells (CellS). *Escherichia coli* bacteria are initially incubated with control cells lacking expression of the target antigen (negative selection). Bacteria that do not bind to control cells are incubated with cells expressing the target antigen on their surface for positive selection. Bacteria bound to antigen‐positive cells after washing (e.g. PBS) are recovered by lysis of the mammalian cells and grown by plating.

We have tested the intimin *E. coli* display system with three different VHH immune libraries, two from dromedaries and one from llamas. Each library was obtained after immunization with antigens of different origin: a purified recombinant bacterial polypeptide (TirM; Salema *et al*., 2013), purified human fibrinogen (Fib; Salema *et al*., 2016a) and cells from the human tumour cell line A431 overexpressing the epidermal growth factor receptor (EGFR; Salema *et al*., 2016a, b). In all cases, the VHH gene segments were amplified from lymphocytes isolated from peripheral blood samples and cloned in frame with the Neae fragment in pNeae2 vector to obtain ~10^7^ independent clones (Fig. 4A).

For the selection of antigen‐specific Nbs, we used magnetic cell sorting (MACS) of *E. coli* bacteria incubated with the corresponding purified antigens labelled with biotin: TirM, Fib and eEGFR‐Fc (the extracellular domain of EGFR fused to human Fc). The MACS procedure for selection is illustrated in Fig. 4B. Induced bacteria are incubated with the biotinylated antigen, washed (e.g. with PBS) to remove the unbound antigen and incubated with a suspension of antibiotin paramagnetic microbeads (Miltenyi Biotec, Bergisch Gladbach, Germany). Next, the mixture is passed through a small ferromagnetic column (mini‐MACS MS; Miltenyi Biotec) held in a magnetic holder, which retains *E. coli* bacteria coated with bound biotinylated antigen and antibiotin microbeads, whereas bacteria with no antigen on their surface are washed out of the column. Elution of bound bacteria is carried out with fresh culture media (e.g. LB) upon removal of the column from the magnet. All steps were carried out at room temperature using mild buffers like PBS and LB, which were sufficient to wash out most non‐specific binders and isolate antigen‐specific clones. Nonetheless, more stringent washing conditions and buffers containing detergents (e.g. Triton X‐100, SDS or deoxycholate) could also be used as *E. coli* with an intact OM can grow in the presence of these detergents (Delcour, 2009).

We chose MACS versus FACS to isolate *E. coli* bacteria bound to the antigen for practical reasons. First, MACS does not require expensive laboratory equipment like the fluorescent cell sorter, facilitating its implementation in most laboratories. Also, processing of libraries is faster in MACS than in FACS, and multiple samples can be run in parallel (e.g. selection of a single library with various antigens, including negative control antigens). In our experiments, we employed a manual system holding up to 8 columns (OctoMACS; Miltenyi Biotec) each with a capacity of at least ~2 × 10^8^ bacteria. Given the size of immune libraries, it was sufficient to load in ~2 × 10^8^ CFU of *E. coli* incubated with the biotinylated antigen (at concentrations from 50 to 200 nM), equivalent to ~0.2 ml of an induced culture at OD_600_ ~1, to have > 10× representation of the immune libraries. Nonetheless, MACS can be automated and is scalable to columns with higher loading capacities (e.g. 2 × 10^9^ bacteria for MACS LS columns; Miltenyi Biotec), thus making screening of large Nb libraries (e.g. naïve) faster and more efficient than FACS. The use of MACS for selections of large naïve scFv libraries by yeast display has also been described (Chao *et al*., 2006).

The number of bacteria eluted from the MACS column in the washed and antigen‐bound fractions can be determined by plating on LB agar as CFU, which provide a quantitative estimation of the enrichment in antigen‐binding clones in every selection cycle. Bacterial colonies grown from the antigen‐bound fraction can be harvested and be frozen for long‐term storage, grown for fluorescence flow cytometry analysis to determine antigen‐binding (e.g. for biotin‐labelled antigens using streptavidin–phycoerythrin, PE) and Nb display levels (e.g. with anti‐myc mAb and fluorophore‐labelled anti‐IgG mouse antibodies) and, if needed, for further cycles of MACS selection. In our experience, the number of cycles required for the isolation of high‐affinity Nb clones varies depending on the library, but usually two rounds of MACS biopanning are sufficient to observe a significant enrichment of antigen‐specific binders from immune libraries allowing isolation of high‐affinity Nb clones with K_D_s in the low nanomolar and subnanomolar range (i.e. 0.2–20 nM; Salema *et al*., 2013, 2016a,b). Upon MACS biopannings, isolation of *E. coli* clones binding the antigen is performed by flow cytometry screening of individual colonies (e.g. 96) randomly picked from the antigen‐bound fraction of the last MACS selection round. The use of flow cytometry for the characterization of Nbs displayed on *E. coli* is discussed later in the text. Lastly, although we have not observed significant differences in growth or loss of viability of *E. coli* bacteria carrying pNeae2 derivatives, we used mild induction conditions (i.e. 0.05 mM IPTG at 30 °C) to minimize potential toxicity effects of Neae‐VHH fusion overexpression from this multicopy vector, which may cause a growth bias of specific clones within the libraries. Similarly, to reduce the potential outgrowth of clones with lower levels of Neae‐VHH expression after each induction–selection cycle, we made preparations of the plasmid pool from antigen‐bound bacteria and used it to transform fresh *E. coli* bacteria for the next round of induction–selection cycle.

### Selection of Nbs by *E. coli* display using selection on live cells (CellS)

Given the potential of Nbs for tumour therapy and *in vivo* tumour imaging, we evaluated *E. coli* display for the direct selection on live cells of Nbs against tumour‐associated cell surface antigens (Scott *et al*., 2012; Tumeh *et al*., 2014; Postow *et al*., 2015; Redman *et al*., 2015). In particular, we performed selection on live cells of Nbs binding human EGFR as a model tumour cell surface antigen (Salema *et al*., 2016a, b). Although MACS is a convenient method to select high‐affinity binders, it relies on the availability of purified antigen, which is not always practical or feasible. Some antigens (e.g. membrane proteins exposed on the surface of tumour cells) may be produced at low yields, have low solubility or may not maintain their native conformation upon purification, all of which limits the construction of immune libraries and the selection strategy. Also, *in vitro* conditions with purified proteins may alter conformational epitopes that could be relevant *in vivo*, leading to selection of Nbs that may not recognize the native antigen on the tumour cell surface. In these situations, it is advantageous to screen Nb libraries directly on live intact tumour cells expressing the antigen, either endogenously or upon transfection. Cell selection (CellS) is also an attractive approach to identify Nbs against novel antigens expressed on the surface of target cells. In the case of phage display, CellS requires experimental optimization to minimize the background binding of phage particles to cells. The more simple phage biopanning procedure involves a depletion step on cells lacking expression of the target antigen, to remove non‐specific phage binders, followed by incubation of the unbound particles with target cells expressing the antigen of interest (Hoogenboom *et al*., 1999; Liu *et al*., 2004; Siva *et al*., 2008). Nonetheless, this simple depletion strategy is usually inefficient with phages and further steps are needed, such as competitive elution with a ligand or mAbs (Figini *et al*., 1998; Beiboer *et al*., 2000; Klimka *et al*., 2000; Veggiani *et al*., 2011), centrifugation of cells through an organic phase (Lipes *et al*., 2008) or masking non‐relevant cell epitopes with soluble Nb fragments (Even‐Desrumeaux *et al*., 2014).

We employed a whole cell‐based experimental strategy (Fig. 4C), from tumour cell immunization to on‐cell selection taking advantage of the excellent specificity of *E. coli* bacteria displaying Nbs to target cells with surface antigens (Piñero‐Lambea *et al*., 2015). We used a library of VHHs obtained by immunizing llamas with A431 human tumour cells that overexpress EGFR (Roovers *et al*., 2007). The VHHs were cloned into pNeae2, induced in *E. coli* bacteria and clones binding EGFR were selected directly on cells using a simple depletion strategy, by combination of a negative selection step on a mouse cell line that does not express human EGFR (named NIH‐3T3 2.2), followed by a positive selection step on cells of the same cell line but transfected to express EGFR (named HER14; Fig. 4C). To screen for binders, we examined by bright‐field light microscopy the adhesion of 96 clones, picked from the second round of CellS, to Her14 (EGFR+) and NIH‐3T3 2.2 (EGFR‐) cells. Clones binding specifically Her14 cells were selected, identifying high‐affinity Nbs that bind specifically EGFR‐Fc by flow cytometry (Salema *et al*., 2016a, b). As high‐affinity binders were predominantly selected during the screening, this indicates that the high levels of display of Nbs on *E. coli* surface and of the target EGFR antigen on the plasma cell membrane of the transfected cell line had no significant avidity effect, which could have resulted in the frequent isolation of low‐affinity binders. The efficiency of selection by *E. coli* display using a simple subtractive CellS suggests that *E. coli* bacteria may have less ‘non‐specific’ binding to cells than phage particles.

### Fluorescence flow cytometry for characterization of Nbs

**Figure 5:**
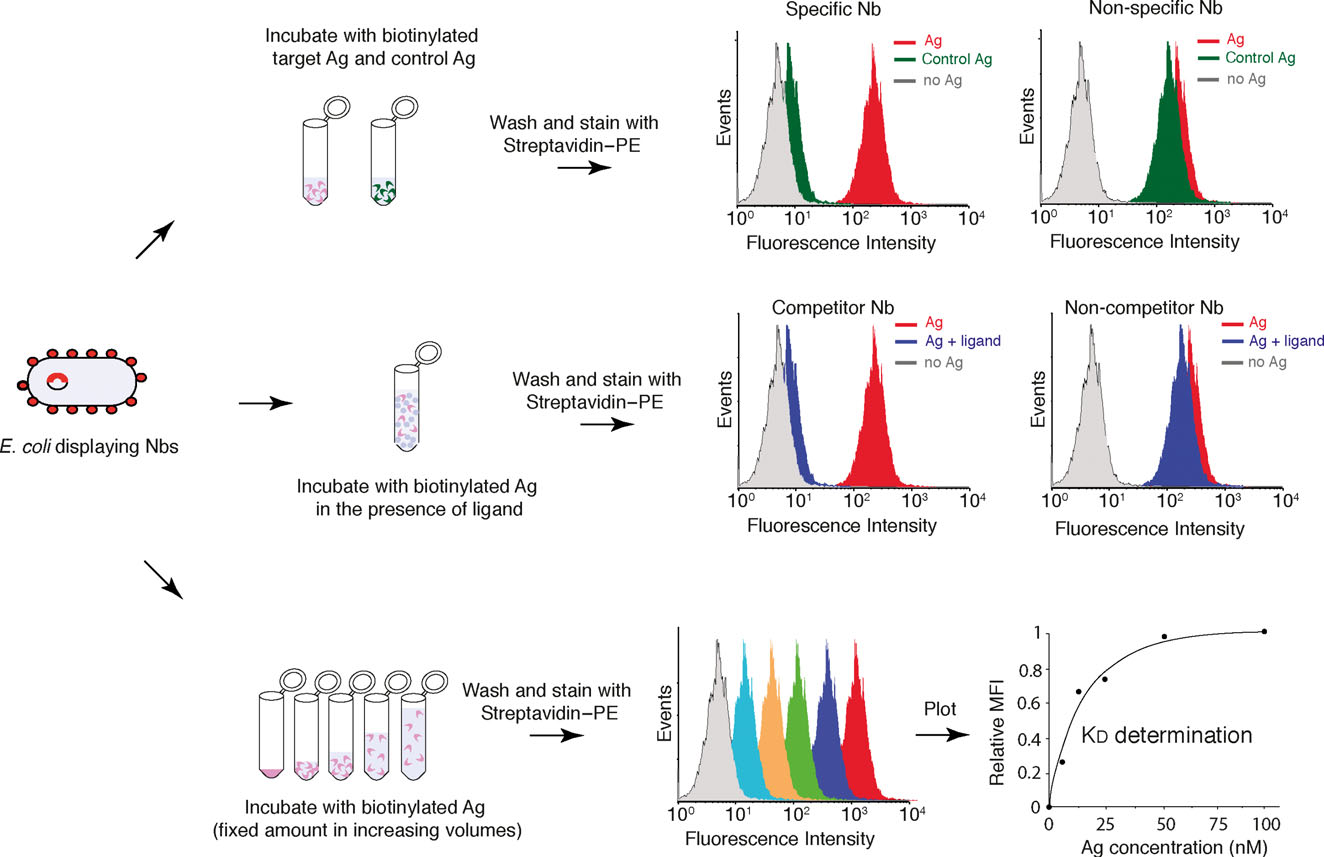
Fluorescence flow cytometry analysis for the characterization of the antigen‐binding properties of nanobodies displayed on *E. coli*. Scheme showing different applications of fluorescence flow cytometry for the characterization of the antigen (Ag)‐binding properties of Nbs displayed on *E. coli*. Top: incubation of bacteria with target and control Ags (e.g. labelled with biotin) to determine binding specificity. Central: incubation of bacteria with labelled Ag in the presence or absence of a ligand (i.e. a molecule binding the Ag) for the identification of Nbs competing or not competing with ligand binding. Bottom: incubation of bacteria with different concentrations of the labelled Ag for the estimation of equilibrium dissociation constant (K_D_) by plotting Ag concentrations versus mean fluorescence intensity (MFI) values. All examples shown use Streptavidin–phycoerythrin (PE) for fluorescent staining of bacteria with bound biotinylated Ag.

The use of fluorescence flow cytometry for the determination of expression levels, antigen binding and estimation of the affinity of selected Nbs is a major advantage of the *E. coli* display system (Fig. 5). Libraries, bacterial pools isolated after selection and individual clones can all be analysed by fluorescence flow cytometry with the anti‐myc‐tag mAb and secondary anti‐mouse IgG antibodies labelled with a fluorophore (e.g. Alexa 488) for the determination of expression/display levels of the Nb on *E. coli* bacteria. Similarly, bacteria can be incubated with different concentrations of the biotinylated antigen used in the selections, or a negative control antigen (e.g. biotinylated BSA), and secondary‐labelled streptavidin (e.g. streptavidin–PE) for the determination of antigen binding and specificity (Fig. 5; Top panel; Salema *et al*., 2013, 2016a,b). Nb display and antigen binding can be analysed simultaneously (double staining) or independently (single staining). Specific binders are identified by flow cytometry screening of individual colonies grown after MACS or CellS. Remarkably, flow cytometry can also be used to analyse binding competition of Nbs with ligands (e.g. EGF ligand EGFR), allowing identification of Nbs that compete (or do not compete) with a ligand (Fig. 5; central panel; Salema *et al*., 2016a, b).

Lastly, this methodology can also be applied to estimate the apparent K_D_ of the Nbs prior to their purification using antigen binding under equilibrium conditions (Fig. 5; bottom panel). This approach has been used to estimate the apparent K_D_ of scFvs on the surface of yeast and demonstrated to be similar to K_D_ values obtained with purified scFv by surface plasmon resonance (SPR; Boder and Wittrup, 2000; Feldhaus *et al*., 2003). We have demonstrated that flow cytometry analysis of *E. coli* bacteria displaying Neae‐VHH fusions gives a good estimation of the apparent K_D_ of Nbs, with values similar to those obtained by SPR with purified Nbs (Salema *et al*., 2013, 2016a,b). A fixed amount of bacteria displaying NVHH fusions (~3 × 10^7^ CFU; ~2 × 10^11^ Nb molecules) are incubated with a fixed amount of biotinylated antigen (~2 pmols; ~1 × 10^12^ antigen molecules) in twofold increasing volumes (e.g. from 0.1 to 10 ml of PBS to reach a final concentration ranging from 20 to 0.2 nM). After 90‐min incubation, bacteria are washed, labelled with Streptavidin–PE and analysed in a flow cytometer. The relative mean fluorescence intensities (MFI) obtained at the different concentrations of antigen are plotted in a curve and the antigen concentration giving 50% MFI is extrapolated to estimate the apparent K_D_ of the Nb.

## Conclusions and future directions

*E. coli* display based on the N‐terminal domain of intimin (Neae) is an effective platform for the surface display of VHH libraries on *E. coli*, allowing selection of high‐affinity Nbs using purified biotinylated antigen with MACS, and on cell selection (CellS) with live mammalian cells displaying the target antigen on their surface. In some circumstances, it might be useful to use FACS in combination with MACS and/or CellS to improve recovery of high‐affinity binders with high level of expression at later stages of the selection, when library diversity is reduced. So far, we have demonstrated that *E. coli* display is suitable for use with Nb immune libraries containing ~10^7^ clones, but larger naïve and synthetic libraries of ~10^9^ clones could also be used as MACS and CellS can be easily scaled up to these bacterial numbers, and suspensions of *E. coli* bacteria at densities of ~10^10^–10^11 ^CFU/ml are handled easily. *Escherichia coli* bacteria with intact OMs can be washed with most common buffers as well as tolerate significant concentrations of detergents (e.g. Triton X‐100, SDS), a marked improvement over the washing conditions tolerated by *E. coli* spheroplasts. The multivalent display and less sticky properties of *E. coli* bacteria compared with bacteriophages facilitates their selection on complex antigenic surfaces, e.g. mammalian cells, tissues and organs with reduced background. Flow cytometry analysis of bacteria is also a major advantage of *E. coli* display, allowing monitoring of the selection process, identification of binders and characterization of their antigen‐binding properties, such as specificity, ligand competition and determination of K_D_. *Escherichia coli* display is likely to be effective not only with camelid VHHs, but also other single domain Abs with similar structural properties, like VNARs from sharks (Dooley *et al*., 2003) and those based on the engineering of human VHs and VLs (van den Beucken *et al*., 2001; Holt *et al*., 2003, 2008; Ignatovich *et al*., 2012). The system might be also suitable for use with larger Ab fragments based on a single polypeptide, like scFvs (Bruce *et al*., 2016), although the tendency of some scFvs to aggregate may hinder their translocation across the OM (Veiga *et al*., 2004). Nonetheless, this property could be advantageous to ‘filter out’ more stable and soluble scFv clones from scFv libraries. Future work will be aimed to demonstrate these potential benefits and applications of *E. coli* display.

## Conflict of Interest

The authors declare that they have no conflict of interest.
